# ATR activity regulates DNA replication and RNA polymerase II transcription during S-phase

**DOI:** 10.1016/j.isci.2026.116663

**Published:** 2026-07-10

**Authors:** Jianming Wang, Sudipta Pathak, Megan Jones, Jingwen Mao, Marco Saponaro

**Affiliations:** 1 Department of Cancer and Genomic Sciences, School of Medical Sciences, College of Medicine and Health, University of Birmingham, Birmingham B15 2TT, UK

**Keywords:** transcription-replication coordination, ATR, transcription deregulation

## Abstract

Transcription and replication both utilize DNA as a template. However, even though conflicts between transcription and replication can induce genome instability, recent data have shown that both coexist temporally and spatially. We have now identified that cells employ multiple mechanisms to control transcription activity in S-phase. Our data show that transcription can be regulated by reducing RNA polymerase II loading on genes or by accumulating it on the promoter and preventing its progress into the gene body; moreover, this can occur on all transcribed genes or only on those replicated at that moment, and all these regulations appear controlled by ATR activity. Finally, we found a correlation between the genes most affected in our system and those with altered expression in patients with cancer with deregulated ATR, potentially linking ATR’s roles in regulating transcription activity during S-phase to phenotypes found in patients with cancer.

## Highlights

• Multiple mechanisms regulate the coordination between transcription and replication
• ATR activity is important to regulate transcription during S-phase
• Ser2-P on RNAPII is a key regulatory step in the process
• Early S-phase replicated genes susceptible to ATRi show defects in ATR deregulated cancers

## Graphical abstract

**Figure:**
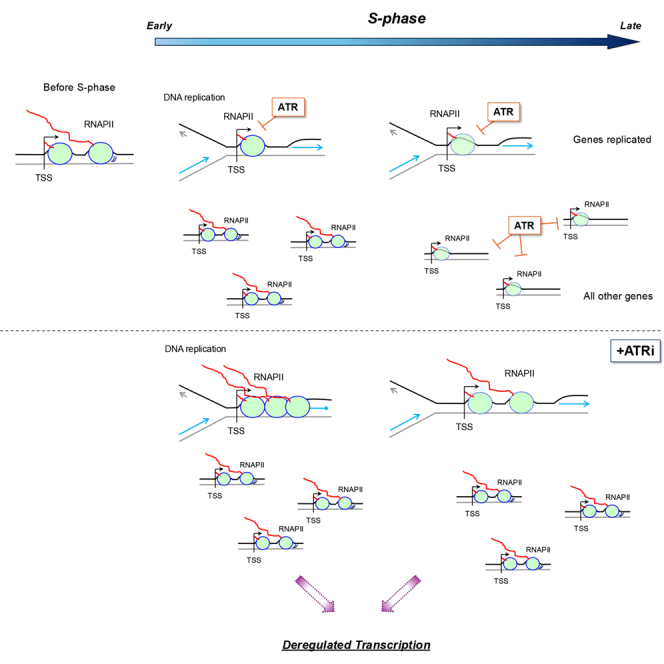

## Teaser

Molecular biology; Epigenetics; Cell biology

## Introduction

RNA transcription and DNA replication are the two processes that use DNA as a substrate. DNA replication mostly occurs during S-phase, with extra S-phase DNA synthesis mainly restricted to instances where DNA replication was affected.^1^^,^^2^ Transcription occurs throughout all stages of the cell cycle, and specifically the transcription of DNA replication factors and histones performed by RNA Polymerase II (RNAPII), occurs during S-phase.^3^^,^^4^^,^^5^ While it is well established that transcription and replication conflict with each other, inducing genome instability,^3^^,^^6^^,^^7^ little is known about if and how cells actively reduce the risks of conflicts. As overlap between the two processes by immunostaining was rare, previous evidence suggested that if a region was replicated, it was not being transcribed simultaneously and vice versa.^8^ However, recent data indicated that transcription was present when genes were replicated, with both processes potentially affecting each other.^2^^,^^7^^,^^9^^,^^10^^,^^11^^,^^12^ Consequently, we hypothesized that there could be specific cellular processes that supported the coexistence of transcription and replication. We recently showed that the DNA checkpoint kinases Ataxia telangiectasia mutated (ATM) and Ataxia telangiectasia and Rad3-related (ATR) preserve genome stability, reducing the formation of genomic rearrangements at transcription-replication collision sites.^7^ To further characterize the role of DNA damage repair and response (DDR) factors in supporting the replication of transcribed regions, we also assessed the inhibition of RAD6, poly-ADP ribose polymerases (PARPs) and PrimPol. This analysis found that ATR was more generally required to support DNA replication during S-phase, as the inhibition of ATR affected replication progression in particular along long transcribed genes. To determine whether ATR activity was also important to regulate transcription at these genes, we performed chromatin-immunoprecipitation sequencing (ChIP-Seq) of RNAPII and its transcriptionally active C-terminal phosphorylated Serine 2 form (Ser2-P). First, we showed that cells use multiple mechanisms during S-phase to control RNAPII activity when genes are replicated. In early S-phase, transcription activity is regulated *in cis* only on genes that are replicated, while later in S-phase there is a global reduction in transcription levels. Importantly, both mechanisms were affected by ATR inhibition, identifying ATR as an important regulator of RNAPII activity during S-phase. Because of the extent of the impact of ATR inhibition on RNAPII transcription and the fact that some of these changes lasted for hours after DNA replication, we investigated whether the deregulation of ATR was associated with a long-lasting impact on the transcription of genes. To do this, first we analyzed RNA-Seq data following ATR inhibition, finding many exons being misincorporated. Next, we analyzed expression data of patients with cancer with loss of function of ATR, finding that across a range of different cancers, deregulation of ATR was invariably associated with lower expression levels of the genes that were the most affected when ATR activity was inhibited in cells.

Altogether, we have expanded our understanding of how DDR factors regulate the interplay between transcription and replication, showing how replication across transcribed regions is supported by the regulation of RNAPII during S-phase. Intriguingly, we found correlations between the phenotypes observed in cells and those observed in patients with cancer with ATR deregulation. This could suggest that the deregulation of RNAPII transcription during S-phase could contribute to some of the phenotypes observed in disease contexts with altered ATR activity.

## Results

### Role of DNA damage response factors in supporting replication across transcribed regions

**Figure 1:**
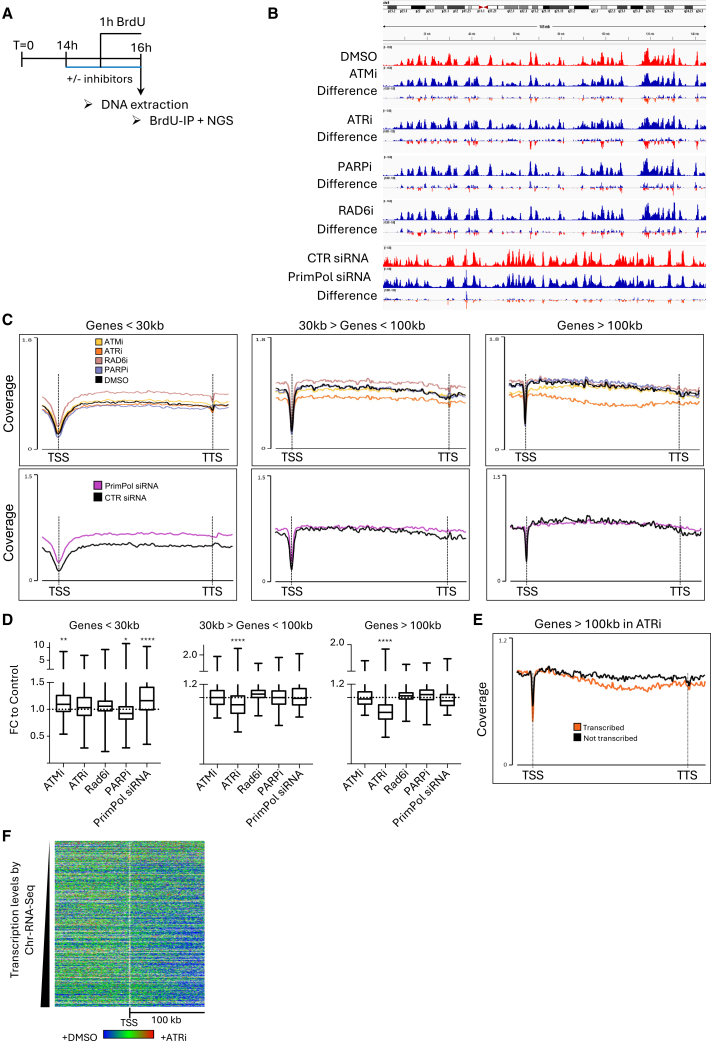
Role of DNA damage response factors in supporting replication across transcribed regions (A) Schematic of the experimental setup, indicating the time points following serum starvation when genomic DNA is extracted, followed by BrdU immunoprecipitation (IP) and next-generation sequencing (NGS): 14 h (G1/S transition) and 16 h (Early S). (B) Chromosome-level view of BrdU incorporation along chromosome 8, with in red the track of the control samples (DMSO or CTR siRNA), and in blue the treated samples (ATMi, ATRi, PARPi, RAD6i, PrimPol siRNA). The Difference profile is the signal of the “treated”—“control,” with signal in blue if higher in the treated and in red if higher in the control. BigWig files viewed on IGV. (C) Average metagene profile for BrdU-Seq in the indicated samples from TSS to TTS along transcribed genes replicated in the first timepoint according to Wang et al.,^2^ with genes clustered according to their lengths. (D) Gene-to-gene fold change (FC) quantifications for the different treatments against their control (DMSO or CTR siRNA), with genes clustered according to their length as in (C); box whiskers plot with the line at the median, and box ± 25% of all the values around the median; Anova analysis of the average BrdU-Seq levels in the different treatments against the average DMSO or siRNA Control samples, for genes of different lengths; *t* test ∗ = *p* value <0.05, ∗∗ = *p* value <0.01, and ∗∗∗∗ = *p* value <0.0001; in all other cases the difference was not statistically significant. (E) Average metagene profile for BrdU-Seq from TSS to TTS in the ATRi-treated sample, specifically along genes >100 kb replicated at that timepoint, separated into transcribed and not transcribed according to the Chr-RNA-Seq from Wang et al.^2^ (F) Ratio heatmap analysis of BrdU-Seq signal in the ATRi vs. DMSO-treated ones on genes >100 kb replicated in that timepoint centered at the TSS ± 100 kb. Genes are ranked according to transcription activity by Chr-RNA-Seq from Wang et al.^2^ Red signal indicates higher BrdU-Seq signal in the ATRi sample, blue signal indicates higher BrdU-Seq signal in the DMSO sample.

Multiple studies have shown how transcription can still be active when genes are replicated,^2^^,^^7^^,^^9^^,^^12^ suggesting that cells may have developed specific processes to control transcription during S-phase to reduce the instances of collisions. However, as not much is known about how this is achieved at the molecular level, to identify factors regulating the coexistence of these two processes, we decided to inhibit or downregulate some DNA damage response factors that are important during S-phase to support DNA replication. BJ-hTERT immortalized fibroblasts were synchronized by serum starvation and released into complete medium to re-enter the cell cycle, as previously described.^2^^,^^7^^,^^13^ 14h after the release, cells at the G1/S transition^2^ were pulsed for 2 h with inhibitors against ATR (4 μM AZD6738), ATM (10 μM KU-55933), Rad6 (10 μM TZ9) or PARP (1 μM Olaparib); alternatively, cells were treated with siRNA trageting PrimPol (Figure S1A). This timepoint was chosen purposely as transcription and replication show the highest overlap in early S-phase.^2^^,^^3^ To monitor DNA replication progression, we added the nucleotide analogue BrdU for the last hour to label DNA synthesis (Figure 1A) followed by BrdU pull-down and sequencing.^2^^,^^7^^,^^13^ All this was integrated with the transcription activity of each gene based on nascent transcription levels, and whether transcription and leading replication forks are moving in the same direction (codirectional) or in opposite directions (head-to-head).^2^

First, we assessed whether treatments affected DNA replication globally and whether we could recapitulate with our genome-wide analysis phenotypes previously observed with other techniques. Comparing BrdU-Seq profiles across whole chromosomes, we identified only small local differences between the different treatments (Figures 1B and S1B). In agreement with previous data, we found that inhibition of RAD6 had very little impact, as RAD6 operated behind the replication forks^14^ (Figures 1B and 1C). ATMi had some impact on BrdU-Seq incorporation levels in short genes, in agreement with previous data showing that the inhibition of ATM had small effects on replication fork rates^15^ (Figures 1C and 1D). Regarding PARPi, we found that it did significantly reduce BrdU levels in short genes replicated at that timepoint and showed higher BrdU incorporation in genes replicated later during S-phase (Figures 1C, 1D, S1B, and S1C); this agreed with faster replication rates,^16^ allowing replication to progress into regions normally replicated later during S-phase (Figure S1C). As PARP inhibition was previously shown to increase transcription-replication conflicts,^17^ we investigated whether PARPi affected the accumulation/persistence of replication forks at stalling/pausing sites, previously identified as BrdU peaks.^7^ PARPi did not affect BrdU peaks that arose in unperturbed conditions (Figure S1D), hence we identified PARPi-specific replication forks stalling/pausing sites. This resulted in 470 PARPi-specific peaks (Figure S1E), 45.5% of which occurred inside transcribed genes replicated in the first two timepoints (16h and 19h) and were enriched for long genes (Figure S1E). These PARPi-specific peaks also presented some BrdU enrichment in control DMSO samples, indicating that PARPi may exacerbate replication fork stalling/pausing at sites that arise at lower frequency in unperturbed conditions (Figure S1E). In agreement with this, when analyzing whether PARPi-specific peaks were associated with increased genome instability, we found that PARPi-specific peaks arose in regions primed with DNA damage in unperturbed cells using γH2AX ChIP-Seq data as a proxy for DNA damage^2^ (Figure S1F). Altogether, we found that PARPi induced a series of novel BrdU peaks rather than increasing stalling/pausing at replication fork stalling/pausing hotspots in unperturbed conditions, enriched in long genes and associated with increased DNA damage. Next, we analyzed the KD of PrimPol, where we observed increased BrdU incorporation levels in shorter genes, indicative of a slowdown of replication fork progression,^7^ in agreement with previous data^18^ (Figures 1C and 1D). However, when we analyzed BrdU levels at BrdU peaks upon the KD of PrimPol, we found a clearer reduction of BrdU levels across most of these sites (Figure S1G), indicating that part of the BrdU signal at BrdU peaks could be PrimPol-dependent repriming. We were able to find BrdU peaks with increased BrdU levels in the PrimPol KD samples, indicative of sites where stalling/pausing of the replication forks increased in the absence of PrimPol, and these presented higher GC content (Figure S1H), in agreement with PrimPol promoting replication restart downstream of G-quadruplexes.^19^ Altogether, our approach recapitulated phenotypes previously observed following the inhibition or KD of these factors, allowing us to investigate the role of these factors in supporting DNA replication across transcribed regions further, providing genomic site information about where they might be required. In this sense, it was ATRi that presented the most profound effect in reducing BrdU incorporation across early replicated genes with the increase of gene length (Figures 1C and 1D). Importantly, this defect appeared dependent on transcription, as long genes that were not transcribed and replicated at the same timepoint did not present a reduction in BrdU levels (Figure 1E). In parallel, when genes >100 kb were sorted by their transcription activity according to Chromatin RNA-Seq,^2^ we found that generally the higher the transcription activity was, the shorter the progression inside the gene was (Figure 1F). At the same time, we found increased incorporation in later replicated genes, especially longer ones (Figure S1I), agreeing with previous data showing that ATR inhibition affected replication timing.^20^ It also identified ATR activity as essential to support the progression of replication forks across transcribed regions, particularly for longer and higher transcribed genes.

### Regulation of RNAPII transcription during genes’ replication

**Figure 2:**
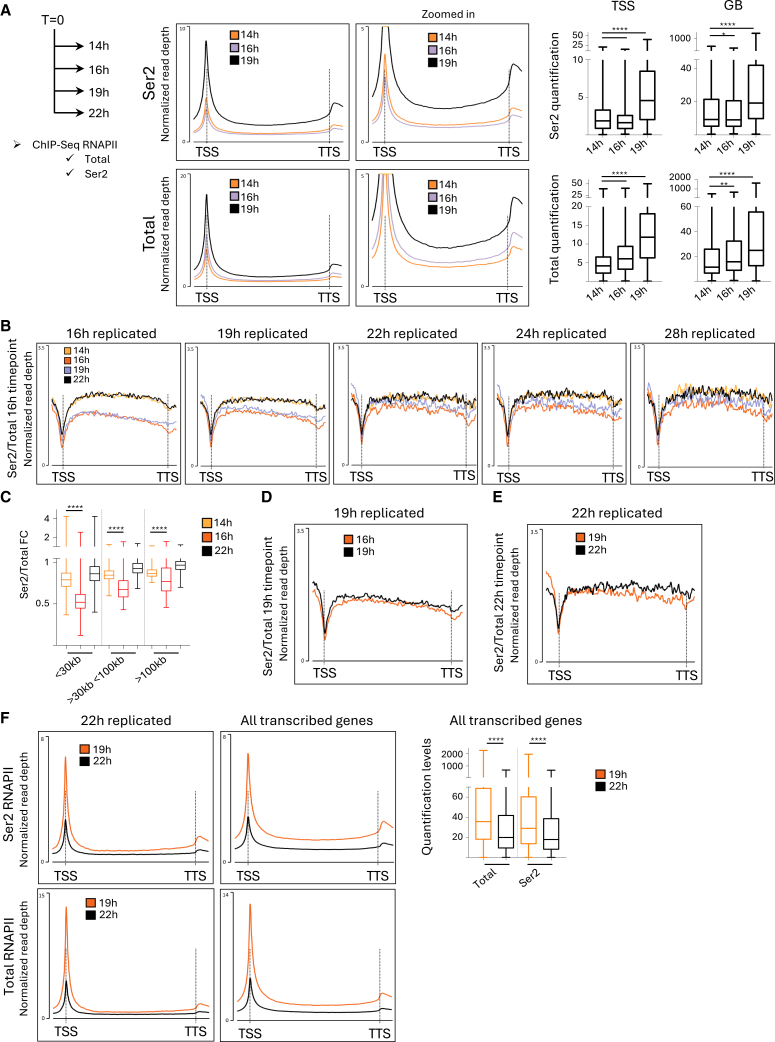
Regulation of RNAPII transcription during genes’ replication (A) Schematic of the experimental setup, indicating the timepoints following serum starvation when ChIP-Seq is performed, and the average metagene profile of Ser2-phosphorylated RNAPII and Total RNAPII on genes replicated at the first timepoint from TSS to TTS, with the 14h, 16h and 19h timepoints, and a zoomed-in view to show better the differences in the gene body. Quantifications of Ser2 and Total RNAPII at the TSS and gene body regions in the 14h, 16h and 19h samples. (B) Average metagene profile of the levels of Ser2 phosphorylated normalized to Total RNAPII from TSS to TTS, with the indicated timepoints and genes clustered by their replication timing according to.^2^ (C) Gene-to-gene quantification of the Ser2/Total RNAPII ratio, at the indicated timepoints, with genes clustered according to their lengths. (D and E) As (B) with the indicated timepoints and genes clustered by their replication timing. (F) As (A) for genes replicated at that timepoint and all transcribed genes, with quantification of the ChIP-Seq levels. Box whiskers plot with the line at the median, and box ± 25% of all the values around the median, Mann-Whitney non-parametric *t* test, ∗ = *p* value <0.05, ∗∗ = *p* value <0.01, ∗∗∗ = *p* value <0.001, and ∗∗∗∗ = *p* value <0.0001; in all other cases the difference was not statistically significant.

To fully determine how ATR supported the replication of transcribed regions, we investigated how RNAPII is regulated in unperturbed conditions, and whether this changed following treatment with ATRi. Previous work showed that RNAPII transcription activity is regulated during S-phase, reducing it temporally when genes are replicated,^2^ and increasing transcription levels for many genes only after their replication.^21^ Nevertheless, the exact mechanistic details on how this was achieved were unknown. Therefore, we performed ChIP-Seq of RNAPII at multiple timepoints during S-phase to determine how RNAPII is regulated, with an additional timepoint at the G1/S transition (14h) to determine RNAPII organization right before DNA replication (Figure 2A). We performed ChIP-Seq of total RNAPII to determine levels and positioning of the RNAPII independently of its post-translational regulations, and of the Serine 2-phosphorylated (Ser2) C-terminal domain (CTD) RNAPII, a post-translational modified form associated with transcription elongation. RNAPII Ser2 ChIP-Seq data across early S-phase replicated genes (16h) showed a reduction of Ser2 levels around the transcription start site (TSS) and in the gene body (GB) compared to the G1/S transition timepoint (14h) (Figure 2A). This reduction was approximately 20% compared to the levels present in G1/S, similar to the reduction in nascent transcription activity,^2^ and was followed by an increase in Ser2 levels by the following timepoint (Figure 2A). Total RNAPII ChIP-Seq data showed a different result, with levels instead increasing across early S-phase transcribed genes, more noticeably across the TSS (Figures 2A and S2A). This would indicate that when genes become replicated, the RNAPII transition into genes is more regulated, with an increase in paused RNAPII and a reduction in Ser2 phosphorylation. At the same time, RNAPII that may escape this pausing and enter the gene, as indicated by the increased total RNAPII in the GB, might not be as proficient as the one normally phosphorylated. A consequence could be that RNAPII might be progressing slower or stall/pause more frequently, leading to a change in the relative distribution of RNAPII between TSS and GB. To assess this, we performed a travel ratio analysis, that is, the ratio between RNAPII ChIP-Seq levels around the TSS divided by its levels in the GB (Figure S2B), previously used to show changes between initiating vs. elongating RNAPII^22^ or in RNAPII elongation rates.^23^ This analysis found that travel ratios were affected for total and Ser2 RNAPII when genes were replicated (16h timepoint), but also that these changes lasted for several hours after replication. To further understand the impact of gene replication on RNAPII regulation, we analyzed Ser2 levels normalized to total RNAPII levels on the genes replicated at that timepoint compared to all other genes. We found that Ser2 levels were greatly affected only on genes replicated at the beginning of the S-phase (16h and 19h, Figure 2B), in agreement with BrdU incorporation levels across these genes (Figure S2C), but not on those replicated later. This would indicate that this regulation of RNAPII occurs only *in cis* on genes replicated at that timepoint and/or in proximity to those replication forks and is not a global change in RNAPII regulation. Moreover, this analysis confirmed that Ser2 levels were deregulated for several hours, and even at the last timepoint we could observe that in the transcription termination regions Ser2 levels were not yet back to pre-replication levels (Figure S2D). When genes were clustered by gene lengths, we found that shorter genes presented the largest variations in their Ser2/Total RNAPII levels (Figure 2C). When we progressed with this analysis across the other timepoints, we did not observe a further reduction in Ser2/Total RNAPII levels at genes replicated at the 19h timepoint (Figure 2D), nor any change on those replicated later (Figure 2E). However, at the 22h timepoint we found a global reduction in RNAPII levels across all transcribed genes (Figure 2F), suggesting perhaps a reduction in transcription activity as cells prepare for mitosis.^4^ All in all, our ChIP-Seq analysis identified two distinct mechanisms regulating transcription activity when genes are replicated, one at the beginning of S-phase, affecting only genes replicated at that timepoint, associated with increased RNAPII pausing at the TSS and affecting Ser2 levels on these genes for many hours even after their replication, and another one global, reducing RNAPII levels across all genes transcribed.

### ATR inhibition affects RNAPII transcription

**Figure 3:**
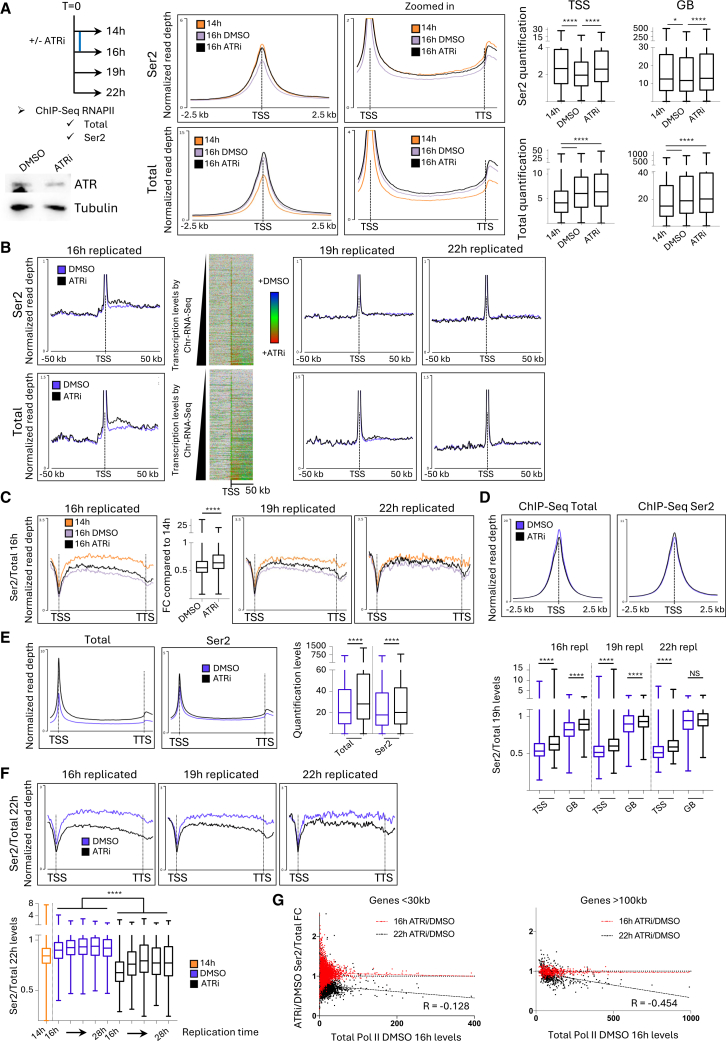
ATR inhibition affected RNAPII transcription (A) Schematic of the experimental set up with indicated the timepoints following serum starvation when ChIP-Seq is performed, with western blot of ATR in cells treated with DMSO or ATRi 4 μM for 2 h, and Tubulin used as loading control; average metagene profile of Ser2-phosphorylated RNAPII and Total RNAPII on genes replicated at the first timepoint, TSS ± 2.5 kb and from TSS to TTS, with the 14h, 16h DMSO and 16h ATRi, and a zoomed in view to show better the differences in the gene body. Quantifications of Ser2 and Total RNAPII at the TSS and gene body regions, in the 14h, 16h DMSO and 16h ATRi samples. (B) Average metagene profile of Ser2 and Total RNAPII ChIP-Seq at genes >100 kb in the 16h DMSO and ATR-treated sample, centered at the TSS ± 50 kb, with a zoom-in to highlight changes to RNAPII levels in the ATRi-treated samples downstream of the TSS. Ratio heatmap analysis of total RNAPII ChIP-Seq from ATRi vs. DMSO-treated samples on genes >100 kb ranked by their transcription activity by Chr-RNA-Seq. Red indicates higher levels in the ATRi sample, blue indicates higher levels in the DMSO sample, green indicates no difference. Average metagene profiles with samples at the same timepoint on genes replicated at the 19h and 22h timepoints. (C) Average metagene profile of the levels of Ser2 phosphorylated normalized to Total RNAPII from TSS to TTS for the indicated samples and genes clustered by their replication timing according to.^2^ Gene-to-gene quantification of the Ser2/Total RNAPII fold change (FC) compared to the 14h timepoint in the 16h DMSO and 16h ATRi samples. (D) Average metagene profile of Total RNAPII and Ser2-phosphorylated RNAPII on genes replicated at that timepoint, TSS ± 2.5 kb. Quantifications of Ser2/Total RNAPII levels at the TSS and gene body regions on genes replicated at the 16h, 19h and 22h timepoint. (E) As (A) on genes replicated at the 22h timepoint, and quantification of Total and Ser2 RNAPII levels on these genes in the DMSO- and ATRi-treated samples. (F) Average metagene profile of the levels of Ser2 phosphorylated normalized to Total RNAPII from TSS to TTS, with the indicated timepoints and genes clustered by their replication timing according to,^2^ with gene-to-gene quantifications in all the timepoints. (G) Scatterplot of the changes in Ser2/Total RNAPII normalized levels in the ATRi samples versus the control DMSO one at the 16h and 22h timepoints by the total RNAPII ChIP-Seq levels at the 16h timepoint, on genes replicated at the 16h timepoint, clustered by gene length. Correlation R values presented on plot, both *p* value <0.0001. Box-whisker plot with the line at the median, and box ± 25% of all the values around the median, Mann-Whitney non-parametric *t* test, ∗ = *p* value <0.05 and ∗∗∗∗ = *p* value <0.0001, NS = not significant; in all other cases the difference was not statistically significant.

When we analyzed the ChIP-Seq data following treatment with ATRi, we observed that Ser2 levels were virtually the same as the pre-replication timepoint, both around the TSS and the GB, and significantly higher than those in the DMSO sample (Figure 3A). At the same time, we observed an increase in total RNAPII levels as in the control DMSO sample (Figure 3A). This would indicate that ATR inhibition affected the Ser2 regulation that occurs at the TSS of genes that become replicated. The increased levels of RNAPII levels inside genes were such that in genes >100 kb we observed an accumulation of RNAPII levels for up to 20-25 kb downstream of the TSS for both total and Ser2 (Figure 3B); this was observed only in the genes replicated in that timepoint and was more pronounced in those with higher transcription levels according to the heatmap (Figure 3B). Considering the BrdU-Seq defect previously described following ATRi (Figure 1F), this would suggest that RNAPII is accumulating behind stalled/slow progressing replication forks. When we compared Ser2/Total normalized RNAPII levels across genes replicated at the different timepoints, we found that ATRi samples presented lower levels of reduction compared to DMSO levels (Figure 3C). To determine whether the increased RNAPII levels inside genes after ATRi treatment led to more collisions between the transcription and replication machineries, and consequently affected replication progression across genes, we determined whether ATRi induced new replication fork stalling/pausing (BrdU peaks). Intriguingly, we identified only 54 ATRi-specific BrdU peaks inside transcribed genes replicated in early S-phase, on average in long genes, and 25 kb away from the TSS or 38 kb from the TTS (Figures S3A and S3B). The number of ATRi-specific peaks was small considering the overall number of genes replicated or compared to the number of PARPi-specific peaks (Figure S1E). Moreover, there were no increased levels of total RNAPII and/or Ser2 in the vicinity of the BrdU peaks, nor in the DMSO or ATRi-treated sample (Figure S3C). This would indicate that replication fork progression inside long genes is not directly affected by more collisions with RNAPII, but that the inability to replicate these genes might be down to intrinsic instability of the replication fork induced by ATRi.^24^ At the same time, it identified ATR activity required in early S-phase to regulate RNAPII transcription when genes were replicated, regulating RNAPII Ser2 phosphorylation to potentially reduce RNAPII levels into genes and support DNA replication.

We continued to monitor the impact of ATRi also after ATRi wash-off, finding that the ChIP-Seq still showed some small differences in the levels of total and Ser2 RNAPII between ATRi and DMSO samples, with a slight persistence of higher Ser2/Total levels across genes, both at the TSS and in the GB, more pronounced over the genes replicated in this and the previous timepoints (Figure 3D). When we analyzed the last timepoint, we found that the ATRi-treated samples presented higher RNAPII levels compared to DMSO treated ones, on genes replicated at that timepoint as well as in all genes (Figures 3E and S3D). Comparing the fold changes in total and Ser2 RNAPII levels, we noticed that Ser2/Total RNAPII levels were this time lower in the ATRi-treated samples, more pronounced for genes replicated at the first timepoint, which was when cells were treated with the inhibitor (16h) (Figure 3F). When we clustered genes by their length, we found that longer genes correlated better with how much Ser2/Total RNAPII normalized levels were affected by RNAPII ChIP-Seq levels in the first timepoint (Figure 3G). We aimed to confirm this increased transcription activity in genes by RT-PCR, analyzing nascent mRNA levels with primers designed across intron-exon junctions in genes replicated in the first timepoint, compared to mature mRNA levels with primers designed across exon-exon junctions. These data generally confirmed higher nascent RNA levels specifically in the last timepoint in the ATRi-treated samples in some of the genes tested (Figure S3F).

Altogether, our data indicated that ATR activity was required at separate moments during S-phase. In early S-phase, ATR supported replication fork stability through transcribed regions and regulated RNAPII levels inside genes, potentially to reduce the risk of collisions between the transcription and replication machineries. Later during S-phase, instead, inhibition of ATR prevents the reduction of RNAPII levels on genes when cells switch off gene transcription and prepare for mitosis.

### Correlation between ATR’s deregulation of RNAPII transcription and cancer patients’ data

**Figure 4:**
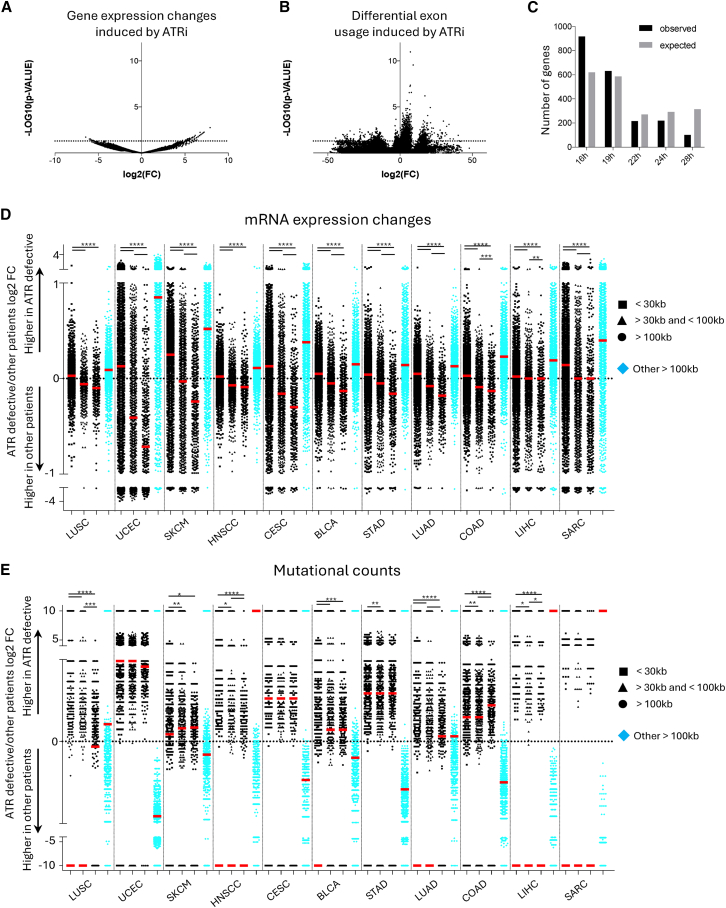
Correlation between ATR’s deregulation of RNAPII transcription and cancer patients’ data (A) Volcano plot of the log2 fold change (FC) in mRNA expression level vs. -LOG10 *p* value, in the ATRi RNA-Seq samples vs. DMSO ones from.^28^ (B) As (A) with the analysis done with single exon usage. (C) Number of observed genes identified with differential exon usage plotted against the expected number; Chi-square analysis for genes replicated in the indicated timepoints, *p* value <0.00001. (D) Log2 fold change (FC) of the mRNA expression levels for the genes replicated in the first timepoint (in black) clustered by gene length, and all other genes >100 kb in cyan, in the listed TCGA cancer studies. Red line at the median. (E) As (D) but for the mutational counts. Mann-Whitney non-parametric *t* test, ∗ = *p* value <0.05, ∗∗ = *p* value <0.01, ∗∗∗ = *p* value <0.001, and ∗∗∗∗ = *p* value <0.0001; in all other cases the difference was not statistically significant.

Previous data showed that ATR can regulate RNAPII transcription in response to DNA damage, especially UV-induced DNA damage, affecting alternative splicing.^25^ In unperturbed conditions, prolonged inhibition of ATR leads to a downregulation of DNA damage repair factors and cell cycle-regulated genes, and an activation of p53 and cGAS/STING pathways, indicative of defective cell cycle progression and accumulation of DNA damage.^26^^,^^27^ Shorter treatments of 2 h with the same ATR inhibitor used in this study do not induce cGAS/STING activation and lead to few changes in gene expression levels.^28^ This is in agreement with data from normal primary mouse cells showing that ATR is dispensable for the expression of DNA initiation replication factors,^29^ indicating that the cell cycle changes induced by ATR inhibition are more a consequence of the DNA damage and replication defects induced by the inhibitors over longer periods of time, rather than ATR directly changing cells’ transcriptional programs. However, given the relevance of Ser2 in regulating transcription elongation and splicing,^30^^,^^31^^,^^32^ we hypothesized that the changes in Ser2/Total phosphorylation levels observed by ATR inhibition could affect correct splicing events. To assess this, we reanalyzed data from^28^ performing an alternative exon usage analysis.^33^ Although ATR inhibition affected only a small set of genes at the mRNA level (Figure 4A), when we analyzed differential exon usage, we observed many exons presenting increased or decreased incorporation in mRNA following ATRi treatment (Figure 4B). This would be indicative of a broader splicing defect rather than specifically a preferential incorporation or exclusion of exons, as observed, for example, in the case of UV-induced DNA damage.^25^^,^^34^ Interestingly, when we assessed the replication timing of the genes affected by the differential exon usage, we observed an enrichment in genes replicated in the first timepoint (Figure 4C), in agreement with our data showing that these genes presented the greatest changes in Ser2/Total RNAPII levels. Indeed, when we performed a gene ontology of the functions encoded by these genes, we found in the top terms for biological processes general cellular processes such as DNA damage response, translation, protein transport, and RNA splicing (Table S1), which will be ubiquitously expressed in cells.

As our data indicated that ATR inhibition affected the transcription of genes replicated at the beginning of the S-phase *in vitro*, we wanted to determine whether changes in the expression of these genes could also be observed *in vivo* in cases with deregulated ATR. To do this, we analyzed RNA-Seq data from The Cancer Genome Atlas (TCGA) PanCancer patients with loss of function of ATR, mutations, and gene deletions, identifying 11 studies with >10 patients with deregulated ATR (Figure S4A). We compared mRNA expression levels of genes replicated in the first timepoint in these patients against all other patients with cancer in the studies. Importantly, these genes are expressed at comparable levels in all cancer types analyzed, and on average at higher levels compared to all other transcribed genes (Figure S4B). We observed consistently across all studies that with the increase in gene length, mRNA levels of genes replicated for the first timepoint were always significantly lower in patients with ATR deregulation (Figure 4D). To determine whether patients with deregulated ATR presented a general defect in the expression of long genes, as a control we compared the expression levels of all other genes >100 kb expressed in our cells but replicated at later timepoints. We found that these were not affected and generally were actually expressed at higher levels in patients with deregulated ATR (Figure 4D). These data indicated that patients with cancer with ATR deregulation present reduced expression levels specifically for the genes replicated at the first timepoint, with the increase in gene length; we found that these genes presented the most pronounced deregulation in Ser2/Total RNAPII levels and altered exon usage, but also were those where we observed a DNA replication defect when ATR was inhibited. Next, to determine whether the changes in expression levels could be related to increased genome instability, we analyzed mutational counts and copy number alterations (deletions and amplifications) across these genes. We found that throughout all cancer types, that early S-phase replicated genes were always less copy number altered in the ATR-deregulated patients compared to the rest of the patients (Figure S4C). At the same time, all the other long genes used as controls for mRNA expression were more prone to copy number alteration in the ATR-deregulated patients (Figure S4C). We often observed increased levels of mutations across early S-phase replicated genes, generally more prominent in the cancers with the largest mRNA expression changes (UCEC, SKCM and CESC) (Figure 4D). This would indicate the mRNA expression levels of genes replicated for the first timepoint are downregulated in patients with cancer with ATR deregulation, not due to changes in their copy number, but potentially linked to an increase in the number of mutations in these genes.

## Discussion

Conflicts between transcription and replication are known to induce DNA damage and drive genome instability.^3^ Nevertheless, recent evidence has highlighted how the two processes of transcription and replication can coexist and occupy the same regions, with direct consequences for replication origin firing, replication fork progression, genome instability and transcription restart following replication.^2^^,^^7^^,^^9^^,^^10^^,^^17^ Consequently, it is conceivable that cells have developed specific mechanisms to regulate the coexistence of transcription and replication. Starting from this assumption, we tested known DNA damage response factors for specific roles in regulating the interplay between transcription and replication.

Our analysis showed that the inhibition of PARP led to the formation of novel stalling/pausing hotspot sites in long transcribed genes, in agreement with a role for PARP in dealing with transcription-replication conflicts.^17^ Interestingly, these sites were already associated with the DNA damage marker γH2AX and presented some levels of BrdU enrichment in unperturbed conditions, indicating that these sites were low-frequency stalling/pausing sites that became exacerbated following PARPi treatment. We also observed an increase in BrdU levels specifically across short transcribed genes, particularly when we used siRNA against PrimPol, indicative of slower replication fork progression as we previously observed following treatments with transcription elongation inhibitors.^7^ Previous work highlighted how PrimPol repriming activity was required to reduce R-loop formation, indicating a role in transcription-replication conflicts.^35^ Inhibition of ATM, PARP and RAD6 presented only mild effects on BrdU-Seq levels inside genes. However, RAD6 had roles across many cellular processes, including chromatin modifications, that ultimately affected transcription elongation.^36^^,^^37^^,^^38^ Therefore, further studies will be required to determine whether potential changes in the BrdU signal observed with the RAD6i were a consequence of direct changes to replication dynamics across transcribed regions, or indirect, due to altered transcription elongation. The major finding was, however, about the role of ATR in supporting DNA replication across transcribed regions and how ATR regulated RNAPII transcription during S-phase.

Through our combined genome-wide analyses, we found that the inhibition of ATR affected cells’ ability to replicate long transcribed genes. However, although we identified specific replication stalling/pausing hotspot sites when we inhibited ATR, these were not associated with an accumulation of RNAPII. This would indicate that ATR activity might be required more generally to support replication fork progression^20^^,^^29^ across regions with further apart origins, rather than specifically stabilizing replication forks in case of collisions with transcription, in agreement with previous data.^7^ While we did not directly measure DNA damage across these regions, the fact that RNAPII transcription showed very little change by the following timepoint (Figure 3D), might suggest that any problem could be transient and/or replication could recover quickly through complimentary mechanisms.^24^ Indeed, this is in agreement with the fact that longer ATRi treatments are required for a significant increase in DNA damage levels,^39^ and can lead only to a small increase in MiDAS levels^40^^,^^41^ associated with replication stress. Equally, ATR inhibition in early S-phase does not induce a significant increase in G2/M DNA synthesis (G-MiDS) levels, which we previously showed to be related to transcribed regions,^2^ like for example occurs with PARPi treatment that induces more transcription-replication conflicts^17^ (Figure S4D).

At the same time, we found that ATR activity regulated RNAPII when genes were replicated. In early S-phase, we found that specifically on replicated genes RNAPII Ser2 phosphorylation is downregulated, with an accumulation of total RNAPII in particular across the TSS. This is akin to an increase in promoter-proximal pausing of RNAPII,^42^ and potentially explains why TSS are hotspots of transcription-replication interactions,^11^ why TSS are under-replicated in S-phase,^2^ and why transcription restarts quickly after replication has entered a gene.^12^ Importantly, this regulation acts mainly on the genes replicated at that timepoint, suggesting that the signal that triggers this change in Ser2 phosphorylation is activated only in proximity to the ongoing replication forks. ATR inhibition disrupts this downregulation of Ser2 phosphorylation, leading ultimately to higher levels of RNAPII inside genes, with an accumulation of RNAPII visible at the 5′-end of long, highly transcribed genes. Replication origins are enriched around the TSS with replication forks proposed to move in the same direction as gene transcription,^43^^,^^44^ and it has already been shown that on long genes RNAPII can be slowed down by the presence of ongoing replication forks.^12^ It is therefore plausible that the accumulation of RNAPII is due to newly initiated transcription events running into affected replication forks while these are still inside genes. This also explains why only the genes replicated in that timepoint presented an accumulation phenotype. In mid S-phase, RNAPII levels were reduced globally, as evident from our RNAPII ChIP-Seq and Chr-RNA-Seq data,^2^ as cells prepare for mitosis where RNAPII is mostly removed from chromatin.^4^ However, while the mid S-phase regulation of RNAPII led to a global reduction in RNAPII levels compared to the early S-phase one that was specific only for the genes replicated, the mid S-phase regulation of RNAPII concerned the loading of RNAPII on genes, while the early S-phase was mainly about RNAPII CTD phosphorylation. Hence, this would indicate that these processes relate to different steps in transcription regulation and consequently, different ATR-regulated target(s). Previously, it was shown that ATR accumulates at the TSS of genes,^45^ although more recent data suggested that ATR recruitment might be more specific to the histone H1 cluster genes.^39^ Much data have identified ATR targets in response to DNA damage and unperturbed conditions,^46^^,^^47^^,^^48^^,^^49^^,^^50^ with transcription being one of the processes particularly regulated. Equally, synthetic lethality studies with ATR inhibitors have identified transcription factors important for the regulation of promoter proximal pausing, as sensitizers to ATR inhibitors.^51^^,^^52^ Future work will determine how ATR regulates RNAPII transcription and whether these sensitivities to ATR inhibitors are related to the deregulations of RNAPII in S-phase identified by our data.

Replication timing and replication origins usage differ greatly between cells.^53^ Nevertheless, many replication origins are shared across cell lines, and some regions are constantly early or late replicated,^53^^,^^54^^,^^55^^,^^56^^,^^57^ linked to their overall transcription activity and chromatin accessibility. Indeed, the genes replicated for the first timepoint in BJ-hTERT cells are expressed on average at high levels across all the cancers analyzed (Figure S4B). Consequently, the regions we have identified as early replicating in BJ-hTERT fibroblasts will be highly likely to replicate early across a wider range of cell types. Indeed, we found that these were the genes where we observed the largest enrichment in deregulated exon usage, with thousands of exons either more or less incorporated in mRNAs in ATRi-treated samples (Figure 4). Intriguingly, we often observed differences in how much genes were affected based on their lengths. This relates to differences in the way gene transcription is regulated on these genes, with transcription generally moving directly from initiation into elongation on shorter genes, with longer genes often having added regulatory steps, but also the fact that longer genes with longer introns tend to have more exons that will need to be appropriately spliced.^58^^,^^59^ Therefore, one possibility that could link the data together is that the deregulation in the correct use of exons may lead to the generation of early stop codons that cause the degradation of the mRNA by non-sense-mediated decay.^60^^,^^61^ Additionally, considering the increased number of mutations inside early S-phase genes in the cancers where we observed the greatest changes in mRNA expression, it could be that cells may actively reduce the expression of these genes because they could be a hotspot for defective replication in the absence of a fully functional ATR. Future studies will determine how much the mis-incorporation of exons drives the changes in gene expression, or whether ATR inhibition, with its impact on the ability to replicate across long transcribed genes, will likewise affect the correct replication of the epigenetic marks on these genes, leading to changes in the chromatin organization that could then affect expression levels.

### Limitations of the study

In this work, we characterized the roles and impact of the inhibition of a series of DNA damage response factors on cells’ ability to replicate across transcribed regions. While this identified specific roles for most of the factors at precise moments in the interplay between transcription and replication, we cannot exclude the presence of compensatory pathways that might have reduced the impact of the inhibition of a single factor. In the future, combinatorial studies should be considered where multiple factors were inhibited together, although this would inevitably increase the complexity of the interpretation of the data, considering the widespread expected impact on replication fork stability progression and stability, but similarly on transcription itself as this work has identified. Moreover, although the mid S-phase deregulation in RNAPII levels was observable in the ATR-inhibited samples, we cannot conclude whether this is a direct or indirect consequence of the ATR inhibition in early S-phase.

## Resource availability

### Lead contact

Further information and requests for resources and reagents should be directed to and will be fulfilled by the Lead Contact, Marco Saponaro, m.saponaro@bham.ac.uk.

### Materials availability

This study did not generate new unique reagents.

### Data and code availability

• Data: The ChIP-Seq data and BrdU-Seq data have been deposited on GEO, with accession numbers: GSE269242 and GSE269289, respectively.
• Code: This paper does not report any original code.
• Any additional information required to reanalyze the data reported in this paper is available from the lead contact upon request.

## Acknowledgments

We thank Genomics Birmingham at the University of Birmingham (UoB) for sequencing our samples and for help and advice with NGS libraries preparation. This work was supported by the University of Birmingham Fellowship to M.S. and grants from 10.13039/100010269Wellcome Trust (202115/Z/16/Z), 10.13039/501100000288Royal Society (RG170246), 10.13039/501100000268BBSRC (BB/S016155/1).

## Author contributions

Conceptualization, M.S.; methodology J.W. and M.S.; investigation, J.W., S.P., M.J., J.M., and M.S.; writing, M.S.; resources, M.S.; funding acquisition, M.S.; supervision, M.S.

## Declaration of interests

The authors declare no competing financial interests.

## STAR★Methods

### Key resources table

**Table:** 

REAGENT or RESOURCE	SOURCE	IDENTIFIER
**Antibodies**		

Rabbit monoclonal anti-Phospho-Histone H3 (Ser10)	Cell Signaling Technology	Cat#3377S
Mouse monoclonal anti-BrdU	Sigma-Aldrich	Cat#B8434; RRID: AB_476811
Total RNAPII (Rpb1 NTD antibody)	Cell Signaling Technology	Cat#14958
Ser2-RPB1	Abcam	Cat#ab26721; RRID: AB_777726
ATR	Santa Cruz	Cat#sc-515173; RRID: AB_2893291
Tubulin	Sigma Aldrich	Cat#T9026; RRID: AB_477593
HRP-linked Horse anti-mouse IgG	Cell Signaling Technology	Cat#7076S

**Chemicals, peptides, and recombinant proteins**		

PARPi (Olaparib)	Selleckchem	Cat#S1060
ATRi (AZD6738)	Selleckchem	Cat#S7693
ATMi (KU-55933)	Selleckchem	Cat#S1092
RAD6i (TZ9)	Cambridge Bioscience	Cat#CAY15964
BrdU	Sigma-Aldrich	Cat#B5002
EdU	Sigma-Aldrich	Cat#900584
Ro 3306	Adooq Bioscience	Cat#A14437
Agencourt AMPure XP	Beckman Coulter	Cat#A63881
Dynabeads Protein A	Thermo Fisher Scientific	Cat#10002D
Fluoroshield with DAPI	GeneTex	Cat#GTX30920
INTERFERin siRNA Transfection Reagent	Polyplus	Cat#409-10

**Critical commercial assays**		

PureLink Genomic DNA Mini Kit	Beckman Coulter	Cat#A63881
DNA Clean & Concentrator-5	Thermo Fisher Scientific	Cat#10002D
RNeasy Mini Kit	GeneTex	Cat#GTX30920
Qubit dsDNA HS Assay Kit	Zymo Research	Cat#D4013
SensiFAST SYBR Lo-ROX Kit	QIAGEN	Cat#74106
Click-iT RNA Alexa Fluor 594 Imaging Kit	Zymo Research	Cat#R1015
NEBNext Ultra™ II DNA Library Prep	New England Biolabs	Cat#E7645
ECL Western Blotting Substrate	Sigma-Aldrich	Cat#DUO92008
SuperScript III Reverse Transcriptase kit	Illumina	Cat#FC-131-1024

**Deposited data**		

RNAPII ChIP-Seq data	This paper	GEO: GSE269242
BrdU-Seq data	This paper	GEO: GSE269289
γH2AX ChIP-Seq data	Wang et al.^2^^,^^13^	GEO: GSE136294
ATRi RNA-Seq data	Feng et al.^28^	GEO: GSE150003

**Experimental models: Cell lines**		

Human immortalized fibroblasts (BJ-hTERT)	ATCC	CRL-4001

**Oligonucleotides**		

siGENOME Non-Targeting siRNA Pool #2	Dharmacon	Cat#D-001206-14-05
siGENOME Human SMARTpool PrimPol	Dharmacon	Cat# M-016804-00
NEBNext Multiplex Oligos for Illumina	New England Biolab	Cat#E6440
See Table S1 for primers used for RT-PCR	N/A	N/A

**Software and algorithms**		

EaSeq	Lerdrup et al.^62^	https://easeq.net
Bowtie 2	Langmead et al.^63^	http://bowtie-bio.sourceforge.net/bowtie2/index.shtml
SAMtools	Li et al.^64^	http://www.htslib.org
STAR	Dobin et al.^65^	https://github.com/alexdobin/STAR
featureCounts	Liao et al.^66^	https://www.rdocumentation.org/packages/Rsubread/versions/1.22.2/topics/featureCounts
MACS2	Zhang et al.^67^	https://github.com/taoliu/MACS
Bedtools	Quinlan et al.^68^	https://bedtools.readthedocs.io/en/latest/
DESeq2	Love et al.^69^	https://link.springer.com/article/10.1186/s13059-014-0550-8
DEXSeq	Li et al.^70^	https://journals.plos.org/plosone/article?id=10.1371/journal.pone.0136653
Prism	GraphPad	Version 7
IGV	Robinson et al.^71^	https://software.broadinstitute.org/software/igv/

### Experimental model and study participant details

#### Cell culture growth and treatments

Human immortalized male fibroblasts (BJ-hTERT cells) were cultured in DMEM (Sigma-Aldrich) supplemented with 10% FBS, 2 mM L-glutamine and penicillin/streptomycin in 5% CO_2_ at 37°C, tested as mycoplasma free. Cell growth, synchronization and labelling of DNA synthesis by BrdU incorporation and sequencing were performed as previously described.^2^^,^^13^ At the indicated timepoints the following drugs were used for the listed time length: ATMi (10 μM, KU-55933 Selleckchem), ATRi (4 μM, AZD6738 Selleckchem), RAD6i (10 μM, TZ9 Cambridge Bisocience), PARPi (1 μM Olaparib Selleckchem). siRNA treatment was performed using INTERFERin siRNA Transfection Reagent (Polyplus) following manufacturer’s protocol. 37.5 nM of siRNAs from Dharmacon were used to target PrimPol (siGENOME Human SMARTpool) as well as a control siRNA (siGENOME RISC-Free Control), respectively, for indicated transfection time. The efficiency of depletion was tested by real time qPCR (see Table S2).

### Method details

#### Primer design and real-time PCR

Primers were designed across exon-intron junctions to assess the levels of nascent pre-mRNA in the gene body far from the TSS, and across two exons to measure mRNA levels (Table S2). RPLP0 was quantified to normalize for the cDNA content in each sample. RT-PCR levels were assessed by quantitative real-time PCR using SensiFast SYBR Lo-ROX kit (Bioline, BIO-94020) and QuantStudio 5 Real-Time PCR System (Thermofisher, A34322) in 96-well PCR plates (Applied Biosystems, 4306737).

#### Genomic datasets alignment and analysis

Paired-end BrdU-seq reads were aligned to the hg38 genome assembly using Bowtie 2 v.2.3.4.2 on the online platform Galaxy,^72^ with BrdU-Seq experiments performed with *N* = 2. Heatmaps were generated with the function “HeatMap” of EaSeq.^62^ In parallel, read coverage profiles were generated also using the computational environment EaSeq version 1.101, normalizing the BrdU-Seq file to the Input DNA file with the function “average”.^62^ BigWig profiles were generated using the function ‘Convert BAM to BigWig’ on Galaxy,^72^ and igvtools “combine data tracks” was used to generate difference profiles on IGV (https://igv.org). For the re-analysis of the RNA-Seq datasets with ATR inhibition,^28^ paired RNA-Seq had reads trimmed for adaptors using the function ‘Trimmomatic’ and mapped with ‘RNA Star’ with transcript based output; followingly, differential expression and differential exon usage analyses were done using the functions ‘DESeq2’ and ‘DEXSeq’, all on Galaxy,^69^^,^^72^^,^^73^ selecting genes with ‘base mean’ >1 and exons with ‘exon base mean’ >0 as transcribed.

Total RNAPII (Rpb1 NTD antibody, Cell Signaling) and Ser2 CTD-phosphorylated RNAPII (Ser2-RPB1, Abcam) ChIP-Seq were performed in cells synchronized as above as previously described.^2^ γH2AX and H2AX ChIP-Seq data were analyzed from^2^ and all ChIP-Seq data were analyzed using EaSeq.^62^

#### Bioinformatic analysis

PARPi-specific and ATRi-specific peaks were called in the two PARPi or the two ATRi BrdU-Seq samples against the two DMSO BrdU-Seq samples, using MACS2 v.2.2.9.1 ^66^ with human genome size and the following parameters: -m 8 30, -p 0.00001 on Galaxy,^72^ as in.^7^ The sites identified represent BrdU peaks present in both treated samples against the control DMSO ones. For the PrimPol GC content analysis, the sequences underlying the peaks coordinates where obtained using the function ‘bedtools getfasta’ on Galaxy, and the function ‘geecee’ to calculate the percentage of GC content across the region.

For the quantifications of ChIP-Seq levels, the function ‘quantify’ was used on Easeq, with windows of from TSS to TTS, or TSS ±500 bp for the TSS levels, and TSS+500bp to TTS for the gene body.

#### cBioPortal analysis

To identify cancers where ATR was more frequently deregulated, we analyzed cBio Cancer Genomic Portal (cBioPortal),^74^ analyzing all The Cancer Genome Atlas (TCGA) PanCancer studies queried for ATR loss of functions: homozygous deletions (homdel), mutations (mut) and low expression levels compared to the average (mRNA expression z-scores relative to all samples (log RNA Seq V2 RSEM, *Z* score < −2)). This identified Lung Squamous Cell Carcinoma (LUSC), Skin Cutaneous Melanoma (SKCM), Uterine Corpus Endometrial Carcinoma (UCEC), Cervical Squamous Cell Carcinoma (CESC), Head and Neck Squamous Cell Carcinoma (HNSCC) Bladder Urothelial Carcinoma (BLCA), Esophageal Adenocarcinoma (ESCA), Stomach Adenocarcinoma (STAD), Ovarian Serous Cystadenocarcinoma (OVCA), Lung Adenocarcinoma (LUAD), Adrenocortical Carcinoma (ACC), Uterine Carcinosarcoma (UCS), Liver Hepatocellular Carcinoma (LIHC), Colorectal Adenocarcinoma (COAD), Cholangiocarcinoma (CHOL), Sarcoma (SARC), as the studies with the largest percentage of patients presenting a deregulation in ATR. ACC, UCS and CHOL were not characterized further because of the low absolute number of cases with ATR loss of function deregulations (respectively 2, 2 and 1). To extract the copy number alterations (CNA) and mutations data we selected the “Comparison/Survival” tab of cBioPortal for each of the PanCancer specific study, selecting “Genomic Alterations” and then either all the “Mutations” or “Copy Number Alterations”. For the mRNA expression we used the tab “mRNA” data. Next, the list of transcribed genes replicated in early S-phase (16h timepoint from^2^) was sorted by gene length, creating three classes of genes: “<30 kb” (3049 genes), “>30 and <100 kb” (1416 genes), “>100 kb” (471 genes). The other class of genes was formed by all other genes >100 kb that were expressed in our system (1601 genes). log2 ratio of the fold change between ATR deregulated patients against all the other cancers for each gene was plotted.

#### Immunofluorescences

Immunofluorescences were performed as previously described.^2^ Briefly, 15 h after release cells were treated with DMSO, ATR inhibitor or PARP inhibitor as above (Sigma-Aldrich) for 1 h, respectively, followed by three washes with warm PBS and cultured again in regular DMEM medium. 24 h after release, 9 μM Ro3306 (Adooq Bioscience) were added to the cells for 16 h to arrest cells in G2. Cells were released into mitosis by vigorous washing and pulsed with 10 μM EdU (Sigma-Aldrich) in fresh pre-warmed medium for 30 min incubated in 5% CO_2_ at 37°C. After one quick wash with ice-cold PBS, cells were fixed and permeabilized for 20 min in PTEMF buffer (20 mM PIPES pH 6.8, 10 mM EGTA, 0.2% Triton X-100, 1 mM MgCl_2_, 4% formaldehyde) at room temperature. Fixed samples were washed 3 times in PBS and stored at 4°C until use. The ‘Click-chemistry’ reaction was performed using Click-iT RNA Alexa Fluor 594 Imaging Kit following manufacturer’s instructions (Thermo Fisher Scientific). Cells were quick washed with 1 mL rinse buffer and blocked for 1 h at room temperature using 10% FBS/PBS (Sigma Aldrich). Cells were washed 3 times in PBS and incubated with 1:2000 diluted rabbit monoclonal anti-Phospho-Histone H3 (Ser10) (Cell Signaling Technology) in 1% FBS/PBS for 1 h at room temperature. Cells were then washed 3 times in PBS and blocked with 1:1000 diluted Alexa Fluor488-conjugated goat anti-rabbit antibody (Thermo Fisher Scientific) in 1%FBS/PBS for 1 h at room temperature. Cells were then washed 3 times in PBS and mounted with a drop of mounting medium with DAPI (GeneTex). Cells were imaged as described above.

#### Western blotting

Cell lysis was prepared by resuspending cells directly in SDS loading buffer followed by sonication using Bioruptor as previously described,^2^ with primary antibodies against ATR (Santa Cruz) and Tubulin (Sigma Aldrich), and HRP-linked Horse anti-mouse IgG (Cell Signaling) as secondary.

### Quantification and statistical analysis

All genomic datasets (BrdU-Seq and ChIP-Seq) were performed with independent biological replicates of *N* = 2. In the case of BrdU peaks, we identified peaks that were significantly present in both the treated samples against both the control files, DMSO for most for samples treated with a specific inhibitor, or CTR siRNA for PrimPol. The nascent and mature mRNA quantifications were performed with independent biological replicates of N ≥ 3, assessed with a two-tailed *t* test. For the other panels, these were analyzed either by Anova against the control samples (DMSO or Control siRNA), or by Mann-Whitney non-parametric *t* test with Graphpad Prism.

## Supplemental information

Document S1. Figures S1–S4

Table S1. Gene ontology analysis of the genes early replicated

Table S2. List of primers used in the paper
