# Supplementary material for: ATR activity regulates DNA replication and RNA polymerase II transcription during S-phase
Source: iScience. 2026 Jul 10;29(8):116663. doi: 10.1016/j.isci.2026.116663 (PMC13380443; doi:10.1016/j.isci.2026.116663)
Supplement: Document S1. Figures S1–S4 [file mmc1.pdf]

iScience, Volume *29*

Supplemental information

ATR activity regulates DNA replication and RNA polymerase II transcription during S-phase

Jianming Wang, Sudipta Pathak, Megan Jones, Jingwen Mao, and Marco Saponaro

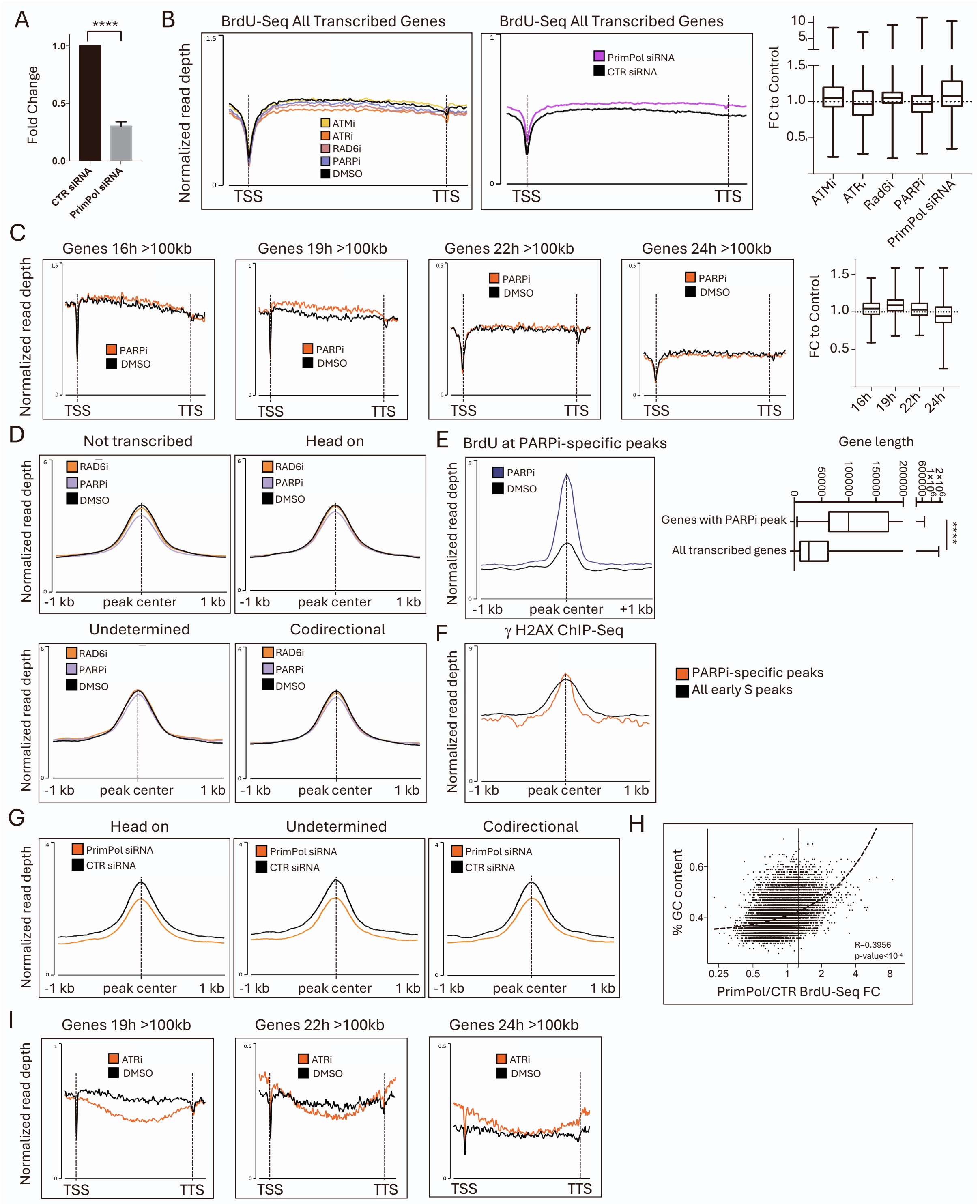

Supplementary Figure 1: Impact of DNA damage repair inhibitors on replication progression.

(A) RT-PCR on CTR and PrimPol siRNA to assess the knockdown efficiency of PrimPol siRNA; n=3, average mean +/- SEM; Two-sided Student t-test, **** = p-value < 0.0001. (B) Average metagene profile of BrdU-Seq from TSS to TTS for all transcribed genes for the indicated samples, with gene-to-gene Fold Change (FC) quantification to their control sample (DMSO or CTR siRNA). (C) As (B) for DMSO and PARPi specifically for genes >100kb

replicated at the indicated timepoints, with gene-to-gene Fold Change (FC) quantification to DMSO control. (D) Average metagene profile of BrdU-Seq signal at the BrdU peaks called in the first timepoint from Rojas et al., [7] +/- 1 kb in the DMSO, RAD6i and PARPi samples, clustered according to reciprocal direction and/or transcribed/not transcribed. (E) As (D) for the PARPi-specific peaks, with gene lengths of the genes with a PARPi peaks against all other transcribed genes. Box whiskers plot with the line at the median, and box +/-25% of all the values around the median, Mann-Whitney non-parametric t-test, **** = p-value < 0.0001. (F) Average metagene profile of γH2AX ChIP-Seq at PARPi-specific peaks and all other peaks identified in that timepoint from [7]. (G) As (D) for BrdU-Seq profiles from PrimPol and CTR siRNA samples. (H) Fold Change (FC) analysis of the BrdU-Seq signal in the PrimPol versus the CTR siRNA samples against the percentage of GC content across the BrdU peak region. Spearman correlation value of 0.3956, p-value $<10^{-4}$. (I) As (C) but in the ATRi and DMSO samples.

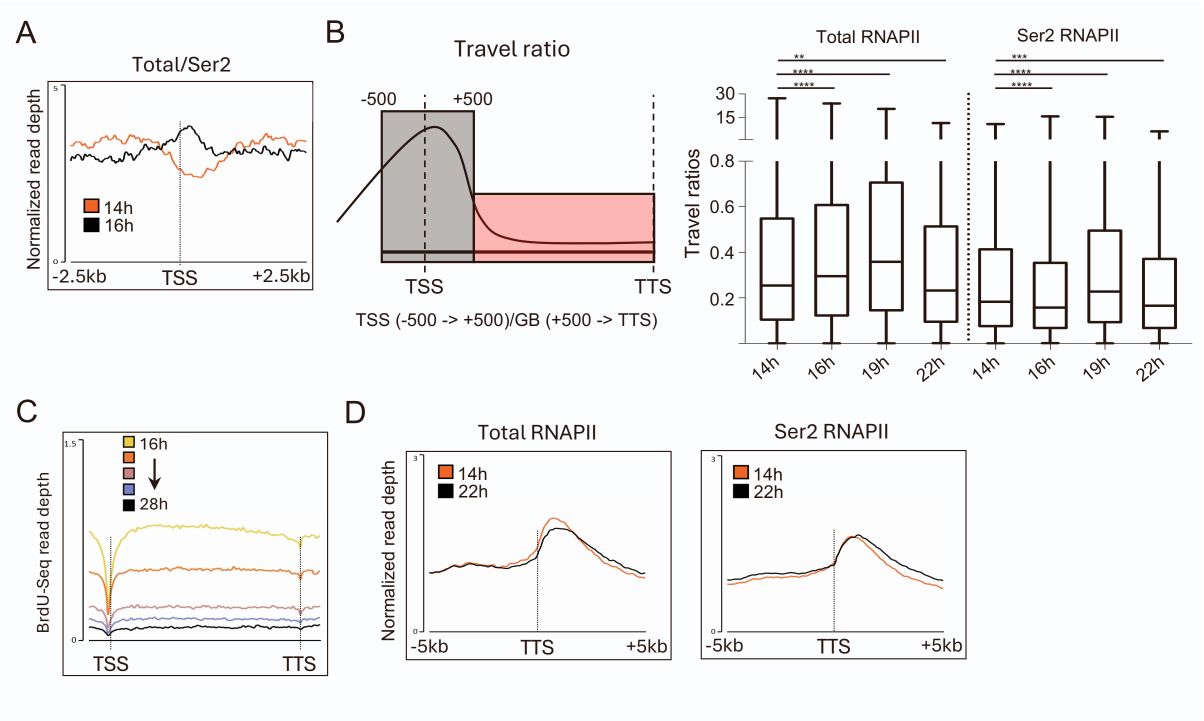

Supplementary Figure 2: regulation of RNAPII transcription when genes are replicated. (A) Average metagene analysis of Total RNAPII against Ser2 RNAPII at the TSS +/- 2.5kb in the indicated timepoints. (B) Schematic of the Travel ratio analysis windows used for the analysis, with Travel ratios plotted for RNAPII and Ser2 in the indicated timepoints compared to the 14h timepoint before replication. Box whiskers plot with the line at the median, and box +/-25% of all the values around the median, Mann-Whitney non-parametric t-test, ** = p-value < 0.01, *** = p-value < 0.001, **** = p-value < 0.0001. (C) Average metagene profile for BrdU-Seq levels from TSS to TTS for genes replicated in all the indicated timepoints. (D) Average metagene profile for Total RNAPII and Ser2 RNAPII at the TTS +/- 5kb of genes replicated in the first timepoint, with the 14h and 22h ChIP-Seq samples.

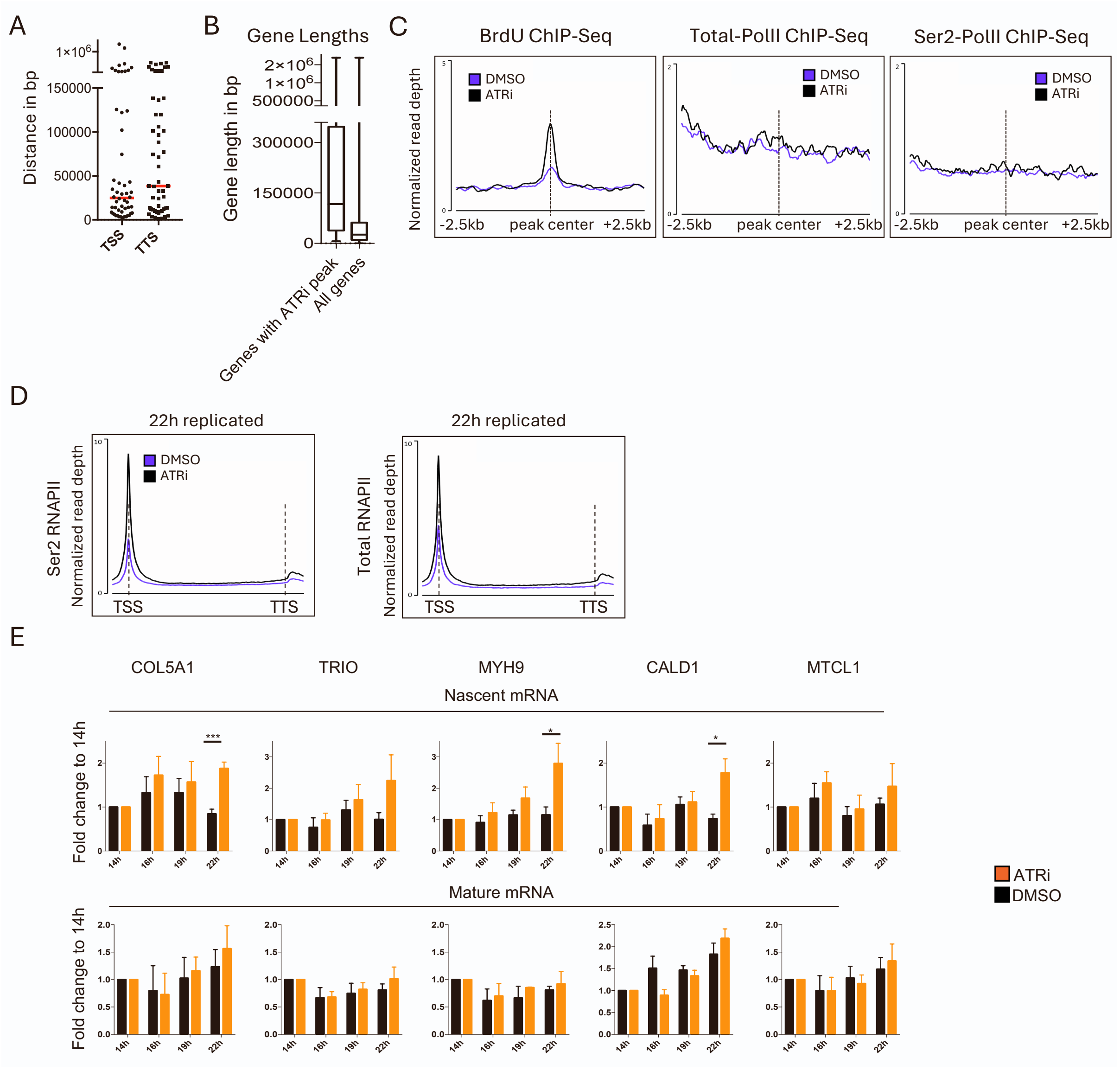

Supplementary Figure 3: ATRi affected RNA Pol II transcription during S-phase

(A) Distance from the ATRi-specific peak inside a transcribed gene to its TSS or the TTS, with red line at the median. (B) Gene lengths of genes with a specific ATRi peak. (C) Average metagene profile of BrdU-Seq, Total and Ser2 RNAPII the ATRi and DMSO 16h samples at the ATRi-specific peaks +/- 2.5kb. (D) Average metagene profiles of Ser2 and Total RNAPII at gene replicated at the 22h timepoint in the 22h DMSO and ATRi samples. (E) RT-PCR analysis of nascent transcription activity and mature mRNA on 5 genes >100kb replicated in the first timepoint (16h), following ATRi or DMSO treatment; n≥3, average mean +/- SEM, two-sided Student t-test, * = p-value < 0.05, ** = p-value < 0.01; in all other cases the difference was not statistically significant.

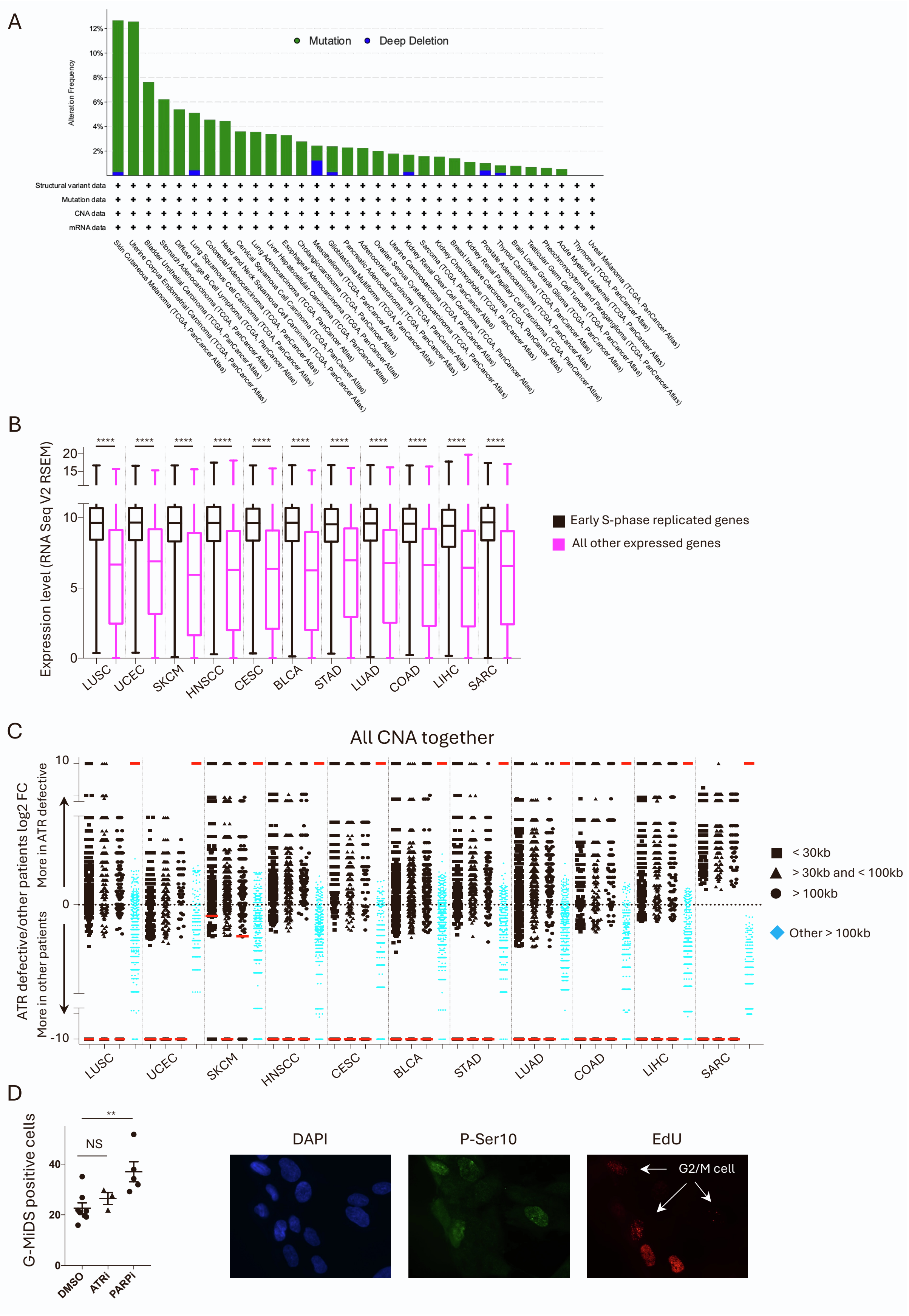

Supplementary Figure 4: Correlation between genes affected by ATRi in cells and in cancer patients

(A) Cancer type summary of the cancers with patients with deregulated ATR, either by mutations or deletions, from cBioPortal. (B) RNA-Seq expression levels for the early S-phase replicated genes and all other transcribed genes in the different cancer types. Box whisker plots with line at the median, and box +/-25% of all the values around the median, Mann-Whitney non-parametric t-test, **** = p-value < 0.0001. (C) Log2 Fold Change (FC) of the copy number alterations (CNA) levels for the genes replicated in the first timepoint (in black) clustered by gene length, and all other genes > 100kb in cyan, in the listed TCGA cancer studies. Red line at the median. (D) Quantification of G-MiDS positive cells following 2h treatment at the 14h timepoints with DMSO, ATRi and PARPi, with representative images of DAPI for nuclear staining, P-Ser10 for phosphorylated Ser10 on histone H3, and EdU for EdU incorporation in G2/M; PARPi is used as a positive control for a reagent that induces more replication stress and more G-MiDS levels. Average mean +/- SEM, n≥3, Anova t-test, ** = p-value < 0.01, NS = not significant.
